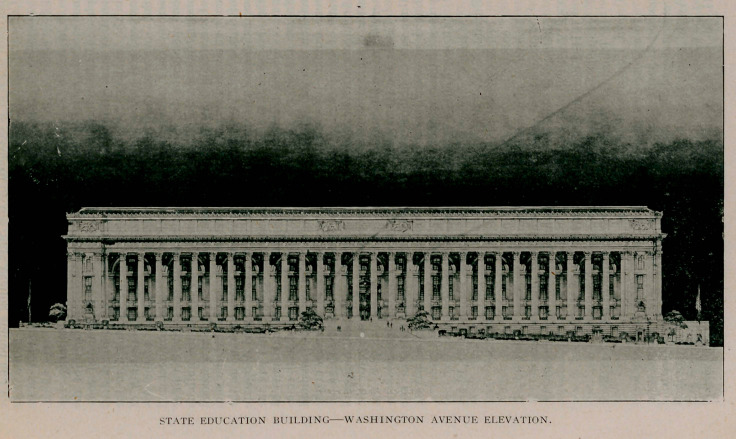# State Educational Building

**Published:** 1909-06

**Authors:** 


A Monthly Review of Medicine and Surgery.

*EDITOR*

WILLIAM WARREN POTTER, M. D.

All communications, whether of a literary or business nature, books for review and exchanges, should be addressed to the editor, 238 DELAWARE AVENUE, BUFFALO, N. Y

VOL. LXIV. JUNE, 1909. No. 11

### State Educational Building

FOR several years past the need for better accommodations for the education department of the state at Albany has been felt. Particularly has this been so since the unification of the University of the State of New York and the department of Public Instruction under the law of five years ago. The officers and employees, about three hundred in all, are widely scattered throughout several floors of the capitol building, besides using other buildings in remote sections of the city. This is inconvenient, delays the transaction of business, and increases the expense of administration.

But more than this, the danger from fire is considerable, in which case the probable loss of life must be reckoned upon, which would be without excuse should it occur. The state library needs more room and better light besides greater convenience.

For these and other reasons not necessary to mention at this time, the commissioner of education, Dr. Andrew S. Draper, took the matter up with the regents, who voted to ask the legislature for authority to construct a separate building, to house and properly accommodate all the interests in charge of the education department. Soon thereafter in conference with prominent members of the legislature a bill was prepared by the Commissioner of Education and introduced in the Senate by Hon. John Raines, providing for the acquisition of a site and the erection of a State Education Building. This bill passed the Legislature with but three opposing votes in both houses and became a law on May 31, 1906, through the approval of Governor Frank W. Higgins.

The Commissioner of Education in his fourth annual report went into the matter in great detail, giving a complete history of building. We extract liberally from the report:

Acting under the authority and direction of this act the Trustees of Public Buildings determined upon the two blocks in the city of Albany bounded by Washington Avenue, Hawk, Elk and Swan Streets with the exception of so much of the more

STATE EDUCATION BUILDING—WASHINGTON AVENUE ELEVATION.

northern block as is occupied by the State heating station and the Cathedral of All Saints. Title was secured to the property in some cases by private purchase and in others by condemnation proceedings.

On August 30, 1906, the Trustees of Public Buildings announced the terms of the first architectural competition for the plans of the State Education Building. This program of competition set forth in detail the size and relation of rooms desired in the building, the plans and elevations to be submitted in the contest, and announced that the Board of Award, consisting of the Governor, the Lieutenant Governor, the Speaker of the Assembly, the Commissioner of Education, the State Architect and one member of the Board of Regents, would select the ten most meritorious designs from the sets submitted, pay to the authors of these designs $500 each and invite them to engage in a second competition. The announcement was further made that the sum of $1,000 would be paid to each architect participating in the second competition and that the Board of Award would designate the first, second and third most meritorious designs and pay the authors of the second and third designs the additional sums of $2,000 and $1,000 respectively, and award the architects' commission for the Education Building to the author of assured responsibility presenting the most meritorious final design at the rate of compensation sustained by the American Institute of Architects after deducting the amounts previously paid him, and after charging him with any proper expense necessary for the services of architectural engineers.

Under the terms of the first architectural competition sixty-three sets of designs were submitted on November 1, 1906. The Board of Award selected the following ten authors as having presented the most meritorious designs:

Allen & Collens, 6 Beacon St., Boston, Mass.

Martin C. Miller & Walter P. R. Pember, Mutual Life Bldg., Buffalo, N. Y.

Pell & Corbett, 31 Union Square, New York City.

George Cary, 184 Delaware Avenue, Buffalo, N. Y.

Palmer & Hornbostel, 63 William Street, New York City.

Wells & Hathaway, 1118 Tremont Bldg., Boston, Mass.

Hedman & Schoen and Goodwin & Jacoby, 302 Broadway, New York City.

J. H. Freedlander, 244 Fifth Avenue, New York City.

Howells & Stokes, 100 William Street, New York City.

P. Thornton Marye and Frederic W. Brown & A. Ten Eyck Brown, Equitable Bldg., Atlanta, Ga.

In a prospectus issued January 10, 1907, these architects were invited to participate in a second competition. This second competition closed on April 1, 1907. After deliberation the Board of Award announced on May 16, 1907, that the design determined to have the highest merit was submitted by Messrs. Palmer and Hornbostel, 63 William Street, New York City, that the second preference was given to the design submitted by Messrs. Howells and Stokes, 100 William Street, New York City and that the third preference was given to the design submitted by Messrs. Miller and Pember, Mutual Life Building, Buffalo. The architects, Messrs. Palmer and Hornbosted, at once began work upon the plans of the building. The preliminary sketches of the plans, elevations and sections were approved by the Board of Award on August 20, 1907. These plans were further revised and approved by the Board of Award on November 27, 1907. The completed working drawings together with the specifications were approved by the Board of Award on February 10, 1908. It is expected that the Trustees of Public Buildings will advertise for bids for the construction of the building by March 15, 1908.

The Board of Award consisted in the first instance of the late Hon. Frank W. Higgins, Governor, and Hon. M. Linn Bruce, Lieutenant Governor, who served until the completion of their terms of office; Hon. James W. Wadsworth, Jr., Speaker of the Assembly; Dr. Andrew S. Draper, Commissioner of Education; the late Hon. George L. Heins, State Architect, who served until his death on September 25, 1907; and Dr. Albert Vander Veer, Regent, who was designated by the Board of Regents in accordance with the statute. Hon. Charles E. Hughes, Governor, and Hon. Lewis S. Chanler, Lieutenant Governor, have served since taking office on January 1, 1907. Hon. Franklin B. Ware, State Architect, has served since his appointment on October 15, 1907. The Speaker of the Assembly, the Commissioner of Education, and Regent Vander Veer have served continuously.

Chapter 578, laws of 1907, the annual supply bill, provides for the construction of the building as follows:

For beginning the construction of the new State Education Building pursuant to chapter 678, laws of 1906, one million five hundred thousand dollars, ($1,500.000), payable on the certificate of the Governor, of which one hundred thousand dollars ($100,-000) shall be available immediately, four hundred thousand dollars ($400,000) on October 1, 1907, and the remainder on March 1, 1908, and from which may also be paid any deficiency in the appropriation made by said chapter for acquiring the site for the building and the expenses incidental thereto. The Trustees of Public Buildings are hereby authorized to enter into contract for the erection and completion of said building, upon terms believed by said trustees to be most advantageous to the State at a total cost of not more than three million five hundred thousand dollars ($3,500,000) for the building exclusive of the site therefor.

The foundation of the building has been laid and the superstructure is under way. When completed the cost, including grounds, will be about $4 500 000 and without doubt the result will be the finest building in the world devoted purely to education purposes. We print a picture elsewhere in this issue, showing the front elevation of the structure on Washington Avenue.

The Commissioner of Education, Dr. Draper is entitled to great credit for working out this difficult problem to a successful conclusion. His well known executive powers were put to a severe test, but they were not found wanting.

---

Dr. Jacob Gould Schurman, president of Cornell University, recently spoke before the Assembly Judiciary Committee in opposition to the antivivisection bills that had been introduced into the legislature. Happily these bills died before the legislature adjourned, but we reproduce the eloquent words of the distinguished educator and commend them to those who agitate the measure annually with such mistaken zeal. Dr. Schurman said in part:

"Who is the genuine humanitarian? Is it the man who wants for himself every variety of animal food, but is so indifferent to the sufferings of his fellow men that he will not allow scientists to conduct their experiments on animals for the relief of those sufferings? The question answers itself. The real humanitarian is the earnest, patient and humane investigator who, with the minimum of pain to animals compatible with his high endeavor to relieve the sufferings of mankind, devotes himself to enlarging our knowledge of the nature and causes of human diseases and discovering the means of their prevention and cure. The man who opposes this beneficent work is either a misguided sentimentalist or an unthinking enemy of his own species.

---

Why is a doctor who wishes to live on Sheridan Road on the same plane with a dog? Because he cannot gain entrance to some of the most "exclusive" of the apartments that line that thoroughfare. This is the condition of affairs, according to the *Chicago Record-Herald,* that has been revealed by the approach of the annual Chicago moving day, May 1.

William M. Webster, who owns the Charlafonte apartments, No. 1545 Sheridan Road, is the pioneer of the movement to exclude physicians and surgeons, and owners and agents of many of the other buildings think so well of his idea that they have agreed not to renew any physicians' leases unless the physicians agree not to receive patients in their homes.

Consequently, it is expected that other quarters will be sought by more than a score of doctors who now live in flats that are satisfactory in every particular.

"Doctors' signs mar the esthetic appearance of a first class apartment building," said Mr. Webster's daughter, explaining for her father why doctors had been barred from their building. "We don't allow dogs or cats or children of a tender age in these apartments and it occurred to father that to make them first class in every respect he might as well place an embargo on physicians while he was at it."

---

Dr. Moses Clegg, bacteriologist of the Bureau of Science at Manila, according to despatches of recent date, has succeeded in cultivating the leprosy bacillus. He has made five separate cultures of the bacillus and carried all of them through five successive generations. Dr. Clegg used the organisms from both living lepers and the bodies of victims of leprosy. He has been equally successful in his cultures with the germs of amebic dysentery, establishing a symbolic relationship between the germs of the two diseases.

The Bureau of Science has prepared a leprosy vaccine, and expects to carry forward a series of experiments for the purpose of establishing a specific treatment for leprosy. Dr. Clegg is the son of a prominent Arkansas physician.

---

Among the paintings at the annual exhibition in the Künstlerhaus at Vienna, one by Adams, showing professor Wertheim and his assistants performing an operation in abdominal surgery, has caused so much comment that Professor Wertheim has written an article on the subject for the *Wiener Medezinische Wochenschrift,* in which he says that "no concessions were made for the painter at the expense of the patient." He confesses that neither gloves nor caps were worn by him or his assistants, "but the omission was not in deference to the wishes of Adams, but because gloves are employed only when the operator fears that he may become infected. And as for the cap, my head is so beautifully bald that I need have no fear that a hair or a speck from it may fall upon the operating field. At operations of this kind my assistants usually wear caps, but in this instance they removed them, and that was the only concession made to the artist." As to masks, the writer says he never uses them. In answer to the criticism that the picture showed a scene which it was needless to exhibit to the public, he writes: "Why so? The paintings of Vereschagin show more horrible situations, but no one objects to them, and while they deal with massacre and torture this picture shows an occurrence which has the well-being of a human sufferer in view. It is well that the public should see us at work; nothing should be secret in the practice of medicine." It were well if other prominent operators would take a similar view.

---

The individual paper drinking cup which Dr. Thomas Darlington, Commissioner of Health, of New York, has been testing this winter has been adopted by the Lackawanna Railroad. Passengers on the Lackawanna Limited have commented on a small nickel-plated device adjacent to the water cooler. Closely nested within a tube are a hundred or more dainty white drinking cups, which, once drawn forth and used, cannot be replaced, but must be discarded or carried away.

These cups, which are in the exact form of a drinking glass, are stiffened by a coating of paraffin, and being manufactured automatically, are untouched by hands until they reach those of the drinker.

Last week a call was issued to fifteen State boards of health to join in a movement to abolish the public train cup. Authorities say that there are few dangers within the reach of the traveling public today so grave from a health point of view as the public drinking cup.

---

John B. Kissinger, of South Bend, Ind., who submitted to the bite of a yellow fever mosquito in the interest of science while in the army in Cuba and for whose relief a bill has been introduced in Congress is now and has been for years almost helpless.

Kissinger was bitten by mosquitoes carrying yellow fever germs and was then treated by the best medical experts in the army. It was supposed he had recovered his health and that as a result of the experiment yellow fever could be guarded against but he later suffered a breakdown, and he is now a physical wreck, unable to use his feet and legs.

Two others died from inoculation, and in each case the Government has given the widow a pension of $100 a month. Kissinger's friends say he is entitled to the same amount.

---

A banquet given at Philadelphia some time ago in honor of Dr. John B. Deaver, the well known surgeon, was unique enough to satisfy the most exacting lover of novelty. All the 113 guests were physicians, each of whom had been operated on by Dr. Deaver either for appendicitis or some other serious disease. The fifteen waiters were laymen who had also been patients of Dr. Deaver.

The waiters were dressed as Red Cross orderlies, and the punch was served in mannikins, each of which had a miniature knife stuck in it at the spot where the incision for an operation had been made. The banquet hall was beautifully decorated with flowers and plants. Many of the guests came from a long distance to pay their respects to the great surgeon, eleven states being represented. The occasion was a delightful one and not one of those present regretted the experience that had qualified him for attendance.

New England has never had a world's fair hence it files a claim through the Boston *Herald,* to the year 1920, in which to celebrate the three hundredth anniversary of the landing of the Pilgrims and the founding of New England. It is proposed to hold a tercentennial exposition in Boston that year, and this early announcement of the fact is made that the whole world may know of the intentions of Boston and New England in the premises. The year 1920 is reserved for the purpose named and, according to the *Herald,* the people of New England will devote the intervening years, in large part, to preparations for an exposition on a scale commensurate with the importance of the event that signaled the birth of the American nation.

---

The fight against tuberculosis is being taken up by fraternal societies in various localities. One of the first to enter the field is the "Modern Woodmen of America." This society several months since, acquired 1,380 acres of land within seven miles of Colorado Springs, and has established thereon a sanatorium, the tent colony plan being employed. The first colony plan was ready for the reception of patients on January 1, 1909, and is equipped to care for 60 patients, to which number admissions must be limited for the present.

The action of this society is commendable and should stimulate similar methods by other organisations of like character.

---

Governor Hughes has signed Senator Davis's bill increasing the term of the medical examiner of Erie County to five years and giving the medical examiner the appointment of his deputy. These changes become effective January 1, 1912, when the terms of the incumbents expire. The present medical examiner, Dr. Danser, and his deputy, Dr. Howland, have performed faithful service during their incumbency, and deserve reappointment when the time shall come for that action.

---

There is a more stringent ordinance now against spitting in public places, says the *Buffalo Express,* April 19. The authorities will make an effort to enforce it. Such an ordinance cannot be enforced without the sympathy and co-operation of citizens generally.

Even in the Board of Aldermen the opinion has been expressed that an anti-spitting ordinance is a mere fad as well as an outrage on the liberties of Plain People. The people must be very plain who do not know that consumption and kindred diseases are spread by spittle. They must be very dense who do not realise that the habit of spitting in public is disgusting to many of their fellow beings.

Perhaps a campaign of education is needed, but in the meantime the authorities should do everything possible to enforce the ordinance. A few examples might make a lasting impression.

---

WITH the going into effect of the new ordinance against spitting in public places, the health department is prepared for a vigorous campaign. The penalty for convictions under this ordinance is arrest and a fine of $2 to $100.

The acting health commissioner, Dr. Fronczak, sent a letter to Superintendent Regan recently notifying him of the provisions of the ordinance and requesting him to see that it is enforced. Circulars are being scattered about the city giving notice of the new rules.

A number of other health provisions went into effect recently. One provides that the Overseer of the Poor may furnish ice to poor families during the hot weather.

## Figures and Tables

**Figure f1:**